# Effect of hydroxychloroquine on COVID-19 prevention in cancer patients undergoing treatment: study protocol for a randomized controlled trial

**DOI:** 10.1186/s13063-021-05292-8

**Published:** 2021-05-19

**Authors:** H. Rahimi, A. Allahyari, S. Ataei Azimi, M. Kamandi, Z. Mozaheb, F. Zemorshidi, M. Khadem-Rezaiyan, A. Bary, M. Seddigh-Shamsi, M. Moeini Nodeh

**Affiliations:** 1 Division of Hematology and Oncology, Department of Internal Medicine, Faculty of Medicine, MUMS, Mashhad, Iran; 2 Department of Internal Medicine, Faculty of Medicine, MUMS, Mashhad, Iran; 3 Department of Neurology, Faculty of Medicine, MUMS, Mashhad, Iran; 4 Department of Community Medicine, Faculty of Medicine, MUMS, Mashhad, Iran; 5 Razavi Hospital, Mashhad, Iran; 6 Current address: Hematology-Oncology Section, Internal Medicine Department, Ghaem Hospital, Ahmadabad Ave, Shariati Sq, Mashhad, Iran

## Abstract

**Objectives:**

In this study, we will investigate the effect of hydroxychloroquine on the prevention of novel coronavirus disease (COVID-19) in cancer patients being treated.

**Trial design:**

This is a two-arm, parallel-group, triple-blind, phase 2–3 randomized controlled trial.

**Participants:**

All patients over the age of 15 years from 5 types of cancer will be included in the study. Patients with acute lymphoid and myeloid leukemias in the first line treated with curative intent, patients with high-grade non-Hodgkin’s lymphoma treated with leukemia regimens, and patients with non-metastatic breast and colon cancer in the first line of treatment will enter the study.

**Intervention and comparator:**

Patients are randomly assigned to two groups: one being given hydroxychloroquine and the other is given placebo. During 2 months of treatment, the two groups will be treated with hydroxychloroquine every other day with a single 200-mg tablet (Amin® Pharmaceutical Company, Isfahan, Iran) or placebo (identical in terms of shape, color, and smell). Patients will be monitored for COVID-19 symptoms during follow-up period.

If any COVID-19-related signs or symptoms occur, they will be examined, thoroughly, investigated with a high resolution computerize tomography (CT) scan of the lungs and nasopharyngeal swab assessed by RT-PCR for SARS-CoV-2 virus. This study will be performed in five centers affiliated to Mashhad University of Medical Sciences, Mashhad, Iran.

**Main outcomes:**

The primary end point of this study is to investigate the incidence of COVID-19 in patients being treated for their cancer and receiving prophylactic Hydroxychloroquine.

**Randomization:**

Randomization will be performed using random permuted blocks. By using online website (www.randomization.com), the randomization sequence will be produced by quadruple blocks. The allocation ratio in intervention and control groups is 1:1.

**Blinding (masking):**

Participants and caregivers do not know whether the patient is in the intervention or the control group. Those assessing the outcomes and data analyzer are also blinded to group assignment.

**Sample size:**

The calculated total sample size is 60 patients, with 30 patients in each group.

**Supplementary Information:**

The online version contains supplementary material available at 10.1186/s13063-021-05292-8.

## Administrative information

**Table:** 

**Title {1}**	Effect of Hydroxychloroquine on COVID-19 prevention in cancer patients undergoing treatment: study protocol for a randomised controlled trial.
**Trial registration {2a and 2b}**	IRCT20200405046958N1 [irct.ir] [registered on 20 April, 2020] https://en.irct.ir/trial/46946
**Protocol version {3}**	Version 6 of 26 March 2021.
**Funding {4}**	This research is funded by the Vice-Chancellor for Research of Mashhad University of Medical Sciences (MUMS).
**Author detail {5a}**	H. Rahimi: Division of Hematology and Oncology, Department of Internal Medicine, Faculty of Medicine, MUMS, Mashhad, Iran. A. Allahyari: Division of Hematology and Oncology, Department of Internal Medicine, Faculty of Medicine, MUMS, Mashhad, Iran. S. A. Azimi: Department of Internal Medicine, Faculty of Medicine, MUMS, Mashhad, Iran. M. Kamandi: Department of Internal Medicine, Faculty of Medicine, MUMS, Mashhad, Iran. Z. Mozaheb: Division of Hematology and Oncology, Department of Internal Medicine, Faculty of Medicine, MUMS, Mashhad, Iran. F. Zemorshidi: Department of Neurology, Faculty of Medicine, MUMS, Mashhad, Iran. M. Khadem-Rezaiyan: Department of Community Medicine, Faculty of Medicine, MUMS, Mashhad, Iran. A. Bary: Hematologist and Medical Oncologist, Razavi Hospital, Mashhad, Iran. M. Seddigh-Shamsi: Department of Internal Medicine, Faculty of Medicine, MUMS, Mashhad, Iran. M. Moeini Nodeh: Department of hematology oncology, Faculty of Medicine, MUMS, Mashhad, Iran. (Current address: hematology-oncology section, Internal Medicine Department, Ghaem Hospital, Ahmadabad Ave, Shariati Sq, Mashhad, Iran.)
**Sponsor {5b}**	Dr. Mohsen Tafaghodi, Vice Chancellor for Research of MUMS, Mashhad, Iran. Email: Ramresearch@mums.ac.ir, Tel: +985138412081
**Role of sponsor {5c}**	This is an investigator-initiated clinical trial. Therefore, the funder played no role in the design of the study and collection, analysis, and interpretation of data and also writing the manuscript.

## Introduction

### Background and rationale {6a}

The emergence of coronavirus disease 2019 (COVID-19) caused by a novel virus from the coronavirus family named SARS-CoV-2 has imposed enormous health and economic problems worldwide.

On the last days of 2019, this disease was reported for the first time in Wuhan, capital of Hubei province, in China, presented with pneumonia. Due to the rapid dissemination of the disease that leads to a pandemic in less than 3 months, there are great concerns about health systems in most countries.

The unknown nature of the virus and the effective methods of prevention and treatment, as well as the high speed of infection spread challenged the scientists. Most of the studies have shown higher incidence and more severity of COVID-19 among cancerous patients [1–7].

COVID-19 imposes great concerns on the patients and their health care providers. The horror of COVID-19 disease that could cause significant morbidity and mortality in immunocompromised patients and lack of specific treatment till now interfere with cancer treatment [8]. Most studies have shown that COVID-19 disease is more severe in cancer patients than others, and the people with cancer have a higher risk of death from COVID-19 disease [6, 9–16].

Some of the most reliable cancer centers in the world have recommended guidelines and advisories for cancer treatment in the COVID-19 era. Most of them have ended up with delay in specific treatment or administration of less toxic regimens. Unfortunately, the consequences of delaying cancer treatment will be determined in the future. Taking rational therapeutic strategies into consideration is of great importance. All cancers must be treated with proper and timely modalities.

As it seems, COVID-19 and its complications are not going to end soon and even it might be a permanent guest of the human being. Nowadays, most of the medical facilities and health care workers capacity are being used by the COVD-19 crisis. Therefore, all of the main health indices such as morbidity and mortality of cancer are being negatively affected.

Several studies have shown the antiviral effect of hydroxychloroquine [17–20]. Hydroxychloroquine (HCQ) is easily available, is easy to take in the form of an oral tablet, and has few monitorable side effects. The drug has no known interaction with chemotherapeutic agents, according to UpToDate®, an evidence-based physician-authored clinical decision support resource and Lexicomp® Drug Interactions [21].

It has been shown that hydroxychloroquine has an inhibitory effect on various viral infections such as adenovirus, influenzas’ virus, and HIV-1. This effect is exerted by preventing the virus from cell entry. Hydroxychloroquine also has good effects on intracellular organisms like malaria and even Coxiella Burnetti. In other studies, in vitro and in vivo antiviral effects of the drug have been shown on the coronavirus family including SARS-CoV-2 [19, 22–26].

### Objectives {7}

Although performing all current approved preventive protocols such as social distancing, hand washing and disinfection, and wearing a face mask in patients have had relatively desirable results, they are not enough to prevent COVID-19 in such susceptible patients.

In this study, we aimed to investigate the effect of the drug hydroxychloroquine on reducing the incidence of COVID-19 disease in cancer patients being treated for their cancer. On the other hand, we decided to compare the rate of COVID-19 infection, the severity of the disease, and its complications in the two groups of cancer patients receiving hydroxychloroquine or placebo.

### Additional consent provisions for collection and use of participant data and biological specimens {26b}

This trial does not involve collecting biological specimens for storage.

### Trial design {8}

This is a phase II–III multicenter two-arm randomized clinical trial designed for cancer patients who have to continue their cancer-specific treatment during COVID-19 pandemic. This trial designed to compare hydroxychloroquine with placebo in COVID-19 disease prevention. The patient allocation ratio is 1:1.

## Methods: participants, interventions, and outcomes

### Study setting {9}

This trial will be performed in two tertiary hospitals, Ghaem and Imam Reza, and three oncology clinics in Mashhad. Referred cancer patients are classified into low-, moderate-, and high-risk groups for COVID-19 involvement. Taking into account statistical considerations, we decided to select the patients just from low- and high-risk groups.

In the low-risk cancers, patients with the most common cancers, breast and colon, were selected. Among the high-risk cancers, patients with acute lymphoblastic or myeloid leukemia and those who have high-grade non-Hodgkin lymphomas treated with leukemia like regimens were selected. These patients would be included if they have below eligibility criteria.

### Eligibility criteria {10}

All patients must have these inclusion criteria:

1. Hematologic malignancies (AML, ALL, high-grade NHL) should be in the first line of treatment with the intention to cure.
2. Included solid tumors should be non-metastatic.
3. Patients older than 15 years

All patients must *not* have bellow criteria:

1. Allergy to hydroxychloroquine
2. Body weight below 35 kg
3. History of retinopathy
4. History of cardiac disease
5. Presence of any sign or symptom of COVID-19 before trial entry
6. Positive serologic screening for SARS-CoV-2 antibodies
7. COVID-19 infection during the first two weeks of trial entry (these patients would be excluded from the study, considering the maximum incubation period of 2 weeks for the infection)
8. Diabetes mellitus
9. Diseases causing immunodeficiency other than cancer
10. Chronic pulmonary disease
11. Immunosuppressive drugs other than cancer chemotherapeutic agents

### Who takes informed consent? {26a}

Eligible patients are selected by the treating physician and given general information about the study. Then, for more familiarity and possible questions and answers, they are referred to the researchers in the clinic. There, after a more detailed introduction of the study in plain language, the informed consent is signed by the patient or his/her legal guardian.

### Intervention {11}

#### Intervention description {11a}

Patients in both study arms will receive a new drug in addition to their anti-cancer treatment. They will receive hydroxychloroquine in the intervention arm, and in the control arm, they will receive placebo. Hydroxychloroquine is administered as one 200-mg tablet every other day. Reasons for using this dose are HCQ long half-life, unknown metabolism, gastrointestinal intolerance in patients undergoing chemotherapy, and minimizing potential side effects while maintaining the efficacy. Participants will take the drug or placebo for 2 months.

Cancer-specific therapies will be chosen according to valid oncologic guidelines by treating oncologist as well as the patient’s opinion. Researchers do not have any role in regimen selection.

Patients are monitored for symptoms and signs of COVID-19 for 10 weeks, including 8 weeks of study intervention and 2 weeks after completion of medication. The symptoms of COVID-19 disease, fever, shortness of breath, cough, myalgia, abdominal pain, and diarrhea, are taught to patients, and if any suspicious symptom occurs, the patient goes to the dedicated clinic. Treating physicians, also, refer the patient to the dedicated clinic whenever they experience suspicious COVID-19 symptoms or any symptoms that are not attributable to the patient’s underlying cancer and its treatment.

Referred patients will be visited by an internal medicine specialist, at a dedicated clinic. For all of these patients, more paraclinical investigations (Additional file 1) are performed.

Based on the symptoms and the result of polymerase chain reaction (PCR) test, the conditions are as follows:

- If a patient has a positive PCR test and he/she has symptoms of COVID-19, he/she is considered to have COVID-19.
- If in a patient the PCR test for 3 consecutive times, each 24–48 h apart, is negative and the patient has typical COVID-19 symptoms, he/she is considered to have COVID-19.
- A patient with 3 negative PCR tests, whose symptoms are not completely typical and have a justifiable cause and disappears in a few days or with specific treatments for other infectious causes, is not considered to have COVID-19.
- It should be noted that asymptomatic patients are not evaluated by PCR.

### Explanation for the choice of comparator {6b}

In the control group, in addition to receiving specific cancer treatment, they are given placebo. All patients use standard preventive health measures including hand hygiene, social distancing, face mask, and isolation.

### Criteria for discontinuing or modifying allocated intervention {11b}

During the study, patients will be monitored for cardiac complications by performing an electrocardiogram (ECG) in the first visit and each chemotherapy session or at least once, every 4 weeks. The most important point in ECG is to calculate corrected QT interval; if the interval is longer than 500 ms, the drug will be discontinued, a drug complication is recorded, and the patient referred to a cardiologist.

At the beginning of the study, a comprehensive history of ophthalmologic problems will be obtained. All patients will be evaluated for any new visual disturbances during the study. In case of new visual disturbances, an examination by an ophthalmologist is warranted and a drug complication is recorded. Since the ocular side effects of HCQ have been reported in long-term use, often after 12 months, periodic examination in asymptomatic patients was not considered.

All patients with hydroxychloroquine drug side effects were excluded from the study in addition to the necessary treatment and compensation measures.

There is not any evidence-based recommendation for baseline cardiac function assessment and ophthalmologic examination in short-term and low dose use of hydroxychloroquine.

Other less common side effects such as hematologic, skin, and gastrointestinal complications are monitored by treating physician. Besides, an Internist is introduced to patients by researcher team just to answer the patients concerns about the study medication or to make an out-of-schedule visit if needed.

### Strategies to improve adherence to intervention {11c}

The drug is taken under the supervision of a nurse in hospitalized patients. The amount of medication used in the out-patient setting is regularly checked, just by history taking, at each chemotherapy visit by the treatment team. Also, a member of the research team checks the regular use of the drug at least once by phone between every two visits of the patients.

### Relevant concomitant care permitted or prohibited during the trial {11d}

All patients must adhere to prevention procedures such as hand hygiene, face mask, social distancing, and isolation throughout the study period.

### Provision for post-trial care {30}

All researchers in this study have occupational liability insurance according to legal requirements in Iran. This insurance will cover all the costs of damages to the patient due to medical malpractice up to 5 years after its occurrence.

### Outcome {12}

The primary endpoint is the comparison of the COVID-19 incidence in the two groups of study. Patients are assessed for the outcome for the entire duration of 8 weeks of drug use and up to 2 weeks after stopping the drug. The secondary endpoint is to evaluate disease severity in both arms. Criteria used to assess disease severity include clinical and paraclinical. Clinically, what shows the severity of the disease is the degree of dyspnea and decreased arterial oxygen saturation. The amount of supplemental oxygen required by the patient and the method of its administration, i.e., the type of mask and invasive or non-invasive ventilation, are also recorded. The best paraclinical criterion for determining the severity of the disease in cancer patients is the extent of pulmonary involvement on lung HRCT scan. Other factors that help include lymphopenia, the rate of increase in inflammatory markers, the length of hospital stay, and the rate of intensive care unit (ICU) stay.

### Participant timeline {13}

Table 1 shows the participant timeline.

**Table 1:**
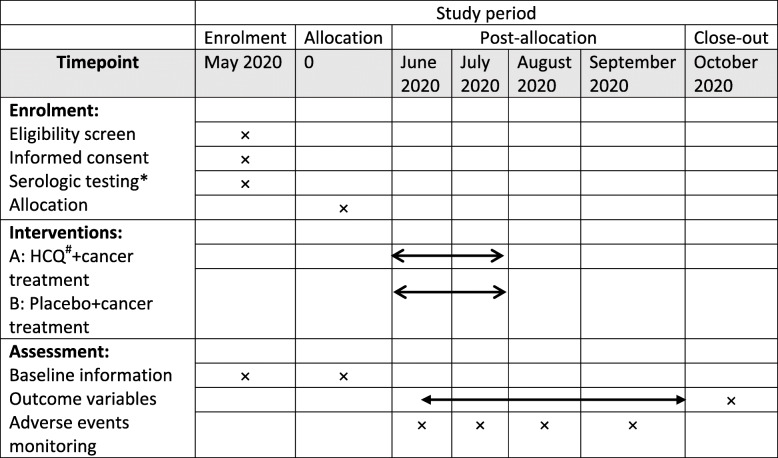
Participant timeline

*Serologic testing consists of SARS-CoV-2-specific antibody by ELISA method

^#^Hydroxychloroquine

The schedule presented in Table 1 was designed from the beginning, but due to the events that occurred, which are described in section {15}, the timeline was extended, the course of which is mentioned in the section {15}.

### Sample size {14}

We considered an assumption of about 40% decrease in the incidence of COVID-19 in cancer patients by administering HCQ. The incidence of COVID-19 in the cancer population has been reported in studies between 1 and 8% [27, 28]. Using the formula for comparing two proportions in G-power version 3.1.9.7 [29], and an alpha error of 0.05, power of 80%, and an allocation ratio of 1:1, the required sample size in each group was estimated as 30 individuals.

### Recruitment {15}

Patients will be recruited from individuals referred to out-patient oncology clinics and in-patient hematology-oncology wards of Ghaem and Imam Reza hospitals, Mashhad, the tertiary and referral centers of hematology and oncology in the east of Iran.

Patients are selected based on inclusion and exclusion criteria by treating physicians and researchers.

Recruitment of this study began in May 2020. Unfortunately, in less than 3 weeks from the start of work in May 2020, recruitment was stopped following the publication of an article against hydroxychloroquine in *The Lancet* journal and WHO statement. Our study was completely stopped for 6 weeks. After retracting the study in *The Lancet* journal, we were able to resume work. Because the results of many studies on hydroxychloroquine were published on those days and often did not support the effect of this drug in treating COVID-19 [30], our recruitment process continued very slowly. Recruitment finally ended in January 2021.

The reason why our study continued after these events was that, according to the WHO recommendation, hydroxychloroquine was excluded from COVID-19 treatment but could be used for prevention in most clinical trial studies [31].

### Assignment of interventions: allocation

#### Sequence generation {16a}

The randomization method was performed base on permuted block randomization. Using the website http://randomization.com/, a random sequence is generated using quadruple blocks.

#### Allocation concealment mechanism {16b}

The sealed, opaque, and sequentially numbered envelopes prepared by an epidemiologist along with packages containing 30 tablets in groups A and B are provided to the treating physicians without specifying which is the drug or the placebo. The content of each random envelop identifies patients’ group, A or B.

#### Implementation {16c}

An epidemiologist will generate an allocation sequence. The treating physician who is blind about the groups selects the first envelope for the first patient and gives the corresponding pill package to the patient.

### Assignment of interventions: blinding

#### Who will be blinded? {17a}

This study is a triple-blind study. The intervention arm is unknown to patients, treating physicians, researchers, and analyzers.

#### Conditions for unblinding {17b}

Whenever a patient experiences any attributable drug side effects, her/his information will be recorded, the intervention would be stopped and necessary care will be applied. Then his/her study group will be revealed to the treating physician.

### Data collection, management, and analysis

#### Data collection methods {18a}

Once the patient enters the study, he or she will be in touch by telephone with the study’s clinic. The clinic provides the necessary training over the phone to the patient to get acquainted with the symptoms of COVID-19. In case of suspicious symptoms or contact with a suspected patient or patient with definite COVID-19, the patient will be invited to the clinic and will be examined clinically and paraclinically (Additional file 1). Besides, all patients at each visit for cancer treatment will be evaluated by their treating physician for signs of COVID-19. If there is any clinical suspicion, the patient is referred to the clinic. The research team was invited to a coordination meeting to review the study protocol. Predictable limitations were discussed and questions were answered. The outcome assessors were trained to reduce any interobserver variations.

#### Plans to promote participant retention and complete follow-up {18b}

Monitoring of patients’ compliance in the study is done in such a way that these patients are either hospitalized or visit every 2 to 3 weeks and therefore are regularly supervised. At the same time, the patient does not have to stay in the study and also does not need to provide any reason to leave it. Explanations are given to the patient about the safety of the drug and additional care that the research team provides would increase patients’ cooperation. There will be no outcome data collected for participants who discontinue or deviate from intervention protocols.

#### Data management {19}

All information collected, including patient’s history and examinations, and their paraclinical information are recorded in the form of papers and electronic files by the assessors. These files are stored in a locked room in the hospital archives for possible future use. The paper files were transferred by the principal investigator to the trial office for data entry. All data were transformed and coded into an SPSS file by an anonymized statistician. This file is also stored in encrypted form on the clinic computer. Before statistical analysis, the SPSS file was checked for data value ranges to find any possible errors.

#### Confidentiality {27}

In the data collection process, each patient is assigned a code. Forms containing the name and code of each patient are available only to the principal researcher of the study and are kept in accordance with the research protocols and the ethics committee. In the process of publishing the results, no names of patients are mentioned.

#### Biological specimens {33}

Biological samples of patients are kept in the Cohort center of Mashhad University of Medical Sciences. This center is equipped for research approved by this university.

Specific COVID-19 antibody test kits were not yet available at the start of this study. Therefore, 5 ml of patients’ blood was taken at the beginning of the study, and after centrifugation, its plasma was kept at minus 20 °C until the antibody testing. From July 2020, when COVID-19-specific antibody testing became available, storage of patients’ plasma samples was eliminated.

### Statistical methods

#### Statistical methods for analyzing primary and secondary outcomes {20a}

Data analysis will be performed using SPSS software version 16 (SPSS Inc., Chicago, IL, USA). Quantitative variables will be described by mean and standard deviation and qualitative variables will be described by frequency and percentage. Quantitative variables between control and intervention groups will be compared by Student’s *T*-test or Mann-Whitney test.

The comparison of qualitative variables between the two groups will be performed by the chi-square test and Fisher’s exact test. For intragroup comparisons, paired *T*-test or Wilcoxon test will be used. All tests will be two-tailed and the significant level will be considered less than 0.05.

#### Methods for additional analyses {20b}

There are no subgroup analyses planned.

#### Methods of analyses to handle protocol non-adherence and statistical methods to handle missing data {20c}

The analysis is based on per-protocol and any dropout or group crossing will be excluded from the final analysis. The probable missing data will not be imputed.

#### Plans to give access {31c}

The datasets used and analyzed during the current study can be made available by the corresponding author upon reasonable request and in agreement with the local research ethic committee.

### Oversight and monitoring

#### Composition and roles of coordinating center and the study team {5d}

This is a multicenter trial designed in Ghaem Hospital, Imam Reza Hospital, Isar oncology clinic, and two other private oncology clinics in Mashhad city (northeast of Iran).

Daily support for the trial is provided by:

Researcher: Trial registration, takes informed consent, supervising the proper implementation of trial and patient health care.

Treating physician: Treats the patient’s cancer, identifies potential recruits, monitors the regular use of medications, and investigates possible side effects.

Clinic physician: Evaluates COVID-19 disease and its severity, manages possible side effects related to the study intervention.

Data manager: Protects blinding, gathers information, and takes care of its confidentiality.

One of the clinicians who is out of the research team knows the group allocation of each patient.

#### Composition of data monitoring committee, its role, and reporting structure {21a}

The data manager monitors the blinding process without having a role to play. The data monitoring committee (DMC) is in contact with patients, continuously, to refer the patients to the clinic in case of drug side effects, non-compliance or symptoms of COVID-19.

#### Description of any interim analyses {21b}

There are no interim analyses planned.

#### Adverse event reporting and harms {22}

In the occurrence of any of the known side effects of the drug, including prolongation of the QT interval and arrhythmia and ocular complication, retinopathy, the information obtained by the treating physician will be reported. This information is recorded by the DMC and if the drug complication is confirmed, the patient will undergo the necessary treatment measures.

#### Frequencies and plan for auditing trial conduct {23}

Due to the short duration of the study and the independence of the researchers, clinic physicians, and treating physicians from each other, there is no program to audit the trial. However, the Institutional Ethics Committee will review the trial conduct, including but not limited to taking informed consent, randomization process, data gathering, and final findings. A dedicated specialist who will be selected by the Vice-Chancellor of Research will also check the research process regularly to ensure that it is in accordance with the approved research proposal.

### Ethics and dissemination

#### Protocol amendments {25}

Important protocol modification (i.e., inclusion and exclusion criteria, outcome, analyses) will be informed to the Ethics Committee of Mashhad University of Medical Sciences and Iranian Registry of Clinical Trials (IRCT).

#### Dissemination policy

##### Dissemination plans {31a}

The results of this study will be published in international peer-reviewed journals. Both positive and negative results will be published. Patients will also be informed of the results of the study if they opt-in to receive the outcome.

## Discussion

This randomized clinical trial is designed to investigate the effect of HCQ on the prevention of COVID-19 in cancer patients. Safety and performance are also monitored.

### Limitations

There are limitations that should be considered during this study. Unknown previous COVID-19 infection of patients entering the study is our greatest concern. Due to our limited resources in Iran, PCR testing has not been performed out-patiently. Therefore, we are satisfied only with the history and serological test of SARS-CoV-2 specific antibodies. Given that the severity of the disease is higher in a cancer patient, it is unlikely that a person has the asymptomatic type of the disease. Another concern is the intolerance to HCQ in patients who experience nausea and vomiting on chemotherapy days. Therefore, the minimum possible dose was selected. The last is the side effects of the drug. Besides choosing the minimum dose, the duration of the study was 2 months. People pay too much attention to COVID-19-related content during the pandemic, which can cause problems. Incorrect information or the results of inconclusive studies sometimes slowed down our trial recruiting, even stopped it for a while.

### Strengths

Health protocols for preventing the transmission of coronavirus among patients in this study will be followed more precisely. Patients will be monitored continuously for COVID-19 during their cancer treatment and appropriate modalities will be started on time, if necessary. If this drug is known to be effective in preventing COVID-19, its most important benefits are inexpensiveness, easily availability, and known toxicity profile.

### Trial status

Recruiting started in April 2020. Following the publication of an article in *The Lancet* against HCQ, which was later retracted, and initial results of SOLIDARITY, WHO’s COVID-19 treatment RCT, we had a long delay in recruiting. Currently (26 March 2021), recruitment is over. The protocol was submitted before the end of recruitment.

## Supplementary Information

**Additional file 1.** Datasheet of patients.

## Abbreviations

MUMS: Mashhad University of Medical Sciences
COVID-19: Coronavirus disease of 2019
IRCT: Iranian Registry of Clinical Trials
HCQ: Hydroxychloroquine
SARS-CoV-2: Severe acute respiratory tract syndrome coronavirus 2
AML: Acute myeloid leukemia
ALL: Acute lymphoblastic leukemia
NHL: Non-Hodgkin lymphoma
ECG: Electrocardiogram
ICU: Intensive Care Unit
ELISA: Enzyme-linked immunoassay
DMC: Data monitoring committee
PCR: Polymerase chain reaction
