# Supplementary material for: Effect of hydroxychloroquine on COVID-19 prevention in cancer patients undergoing treatment: study protocol for a randomized controlled trial
Source: Trials. 2021 May 19;22:349. doi: 10.1186/s13063-021-05292-8 (PMC8131879; doi:10.1186/s13063-021-05292-8)
Supplement: Supplementary file 1 — Additional file 1. Datasheet of patients. [file 13063_2021_5292_MOESM1_ESM.docx]

**Appendix 1.** Datasheet of patients.

| Patient identity | Name: …………………. | Code: ………………… | Study arm: …………… | |
| --- | --- | --- | --- | --- |
| Cancer-related information | Cancer type: ………………. | Last Chemotherapy cycle (date): ……………… | | |
|  | Treatment location:  Hospital, in-ward □ Hospital, out-ward □ Out-patient clinic □ | | | |
| Study-related information | Date of enrollment: ……… | Drug attachment: complete □ Irregular □ Discontinued □ | | |
|  | Suspicious contact (with COVID-19 patient): Yes □ No □ | | | |
| COVID-19 Symptoms | Fever: Yes □ No □ | Dyspnea: Yes □ No □ | Cough: Yes □ No □ | |
|  | Myalgia: Yes □ No □ | Abdominal pain: Yes □ No □ Diarrhea: Yes □ No □ | | |
|  | Vomiting: Yes □ No □ | Other: ……………… | | |
| COVID-19 Signs | Fever: ……….... | Respiratory Rate: ……… | Heart Rate: ………… | |
|  | O2Sat (room air): …...... | Others: …………… | | |
|  | Supplementary O2: Nasal canula □ Ventury mask □ Reserve Bag □ NIV* □ MV^ □ | | | |
| Paraclinical Findings | WBC: ……. /µL | ALC**: ……. / µL | | PLT: ……… / µL |
|  | CRP: ………. | LDH: …………. | |  |
|  | HRCT Score^^: ………. /24 | SARS-CoV-2 RT-PCR: Positive □ Negative □ | | |
| COVID-19 outcome | Home Isolation only □ Hospitalization □ (Duration ………. Day(s)) ICU admission □ (Duration ………. Day(s)) Recovery □ Death □ | | | |

* Non-invasive Mechanical Ventilation

^ Mechanical Ventilation

** Absolute Lymphocyte Count

^^ According to the American College of Radiology guideline.
